# The use of octreotide in pediatric patients: Practical applications for gastrointestinal disorders and beyond: A narrative review

**DOI:** 10.1002/ncp.11348

**Published:** 2025-07-09

**Authors:** Bailey Dunn, Megan Foxe, Kathy Hunter Sprott, Jessica E. Hook, Candi Jump

**Affiliations:** ^1^ Department of Pediatrics Medical University of South Carolina Charleston South Carolina USA; ^2^ Shawn Jenkins Children's Hospital Medical University of South Carolina Charleston South Carolina USA; ^3^ Shawn Jenkins Children's Hospital, College of Pharmacy Medical University of South Carolina Charleston South Carolina USA; ^4^ Department of Pediatrics, Division of Pediatric Cardiology Medical University of South Carolina Charleston South Carolina USA; ^5^ Department of Pediatrics, Division of Pediatric Gastroenterology, Hepatology, and Nutrition Medical University of South Carolina Charleston South Carolina USA

**Keywords:** gastrointestinal bleeding, lymphatic disorders, octreotide, pancreatitis, secretory diarrhea, somatostatin

## Abstract

Somatostatin is a naturally occurring polypeptide hormone that exerts its effect on the gastrointestinal tract by reducing exocrine and endocrine secretion, resulting in decreased motility, gastric emptying, splanchnic blood flow, fat absorption, lymphatic flow, and gallbladder contraction. Octreotide is a synthetic somatostatin analogue that has a variety of clinical applications in the gastrointestinal tract, including in the treatment of gastrointestinal bleeding, motility disorders, lymphatic disorders, pancreatic disorders, and high‐output states. Clinicians may hesitate to use octreotide because of its potential side effects and the lack of robust pediatric data. Here we describe potential side effects of the drug and review the use of octreotide in the above pediatric indications.

## INTRODUCTION

Octreotide is a synthetic somatostatin analogue with a broad range of clinical applications, largely due in part to its action in a variety of organ systems. The prolonged half‐life of octreotide compared with the naturally occurring peptide somatostatin allows for its use as a therapeutic agent.^1^ The aim of this article is to provide a review of the clinical application of octreotide in pediatric disease states.

## OCTREOTIDE MECHANISM OF ACTION

Somatostatin is a naturally occurring tetradecapeptide with a high concentration of receptors found in the central nervous system, hypothalamus, pituitary gland, pancreas, and gastrointestinal (GI) tract. In general, somatostatin functions as an inhibitory peptide via endocrine, paracrine, or autocrine mechanisms or as a neurotransmitter. The main targets of somatostatin are the endocrine system and the GI tract.^2^ In the hypothalamus, somatostatin inhibits the release of growth hormone (GH) and thyrotropin (TSH). In the pancreas and GI tract, it reduces endocrine and exocrine secretion.

The therapeutic use of somatostatin is limited by its short half‐life, only 2–3 min, which makes continuous intravenous administration mandatory.^3^ Octreotide, a synthetic analogue of somatostatin, has the same therapeutic properties with a longer half‐life, 1–2 h, and greater potency, which makes it more clinically useful.^4^

Octreotide binds to somatostatin receptors, specifically G protein receptors, that inhibit adenylate cyclase and reduce the production of cyclic‐AMP.^1^ Physiologically, octreotide prevents the secretion of vasoactive intestinal peptide (VIP), secretin, insulin, glucagon, motilin, gastrin, and pancreatic polypeptide. In addition, release of GH, insulin‐like growth factor‐1, luteinizing hormone, and TSH are suppressed.^2^ In the GI tract, this results in decreased motility, gastric emptying, splanchnic blood flow, fat absorption, lymphatic flow, and gallbladder contraction.^5^

Current US Food and Drug Administration (FDA)–approved indications for the use of octreotide in adults include acromegaly, severe diarrhea and/or flushing secondary to carcinoid tumors, and severe diarrhea secondary to VIP‐secreting tumors. Octreotide has also been used off‐label for several other endocrine, digestive, and oncologic indications.^6^ In pediatric patients, octreotide is primarily used off‐label for its effects in GI bleeding, chylothorax, GI motility, pancreatitis, and high secretory output.^7^

### Adverse effects

Common adverse effects (>10%) reported in the prescribing information for octreotide include bradycardia, hyperglycemia, abdominal discomfort with nausea/diarrhea, hypothyroidism, and cholelithiasis. Adverse effects in children are like those seen in the adult population; however, several studies have noted some additional safety concerns.

A large study of 428 critically ill hospitalized infants who received 490 courses of octreotide reported a relatively low risk of hyperglycemia and hypoglycemia (1 out of 1000 and 3 out of 1000 infant‐days, respectively).^5^ There was a 12% incidence of hypotension requiring initiation of vasopressors. Finally, the authors reported an incidence of medical necrotizing enterocolitis (NEC) of 2% and surgical NEC in 0.2% of the infants studied. Ultimately, it was concluded that the rate of NEC was similar to that reported in the literature and causation was not established with laboratory abnormalities or hypotension.^5^

A study of children treated with octreotide for congenital hyperinsulinism noted transient elevation of liver enzymes in 46.6% of patients with resolution within 4 to 8 weeks, despite continued octreotide. Gallbladder sludge or stones were noted via ultrasonography in 32.1% of patients, with half of the patients with gallstones having resolution of stones with initiation of ursodeoxycholic acid.^8^

A concern of particular importance in children is octreotide's potential impact on linear growth given suppression of GH. In a case series describing the use of long‐acting octreotide for intractable GI bleeding,^9^ a single patient was diagnosed with GH deficiency in the setting of reduced height velocity and no response to a GH stimulation test. After initiation of GH therapy, growth and development were reported to normalize. In another study that reported 28 patients with congenital hyperinsulinemia receiving long‐term octreotide, three patients were diagnosed with short stature, but none had abnormal GH stimulation testing.^8^ Close monitoring of growth and evaluation of short stature seems reasonable in those requiring long‐term therapy with octreotide.

Cases of cardiovascular side effects have been reported with octreotide. One such effect is Q‐T interval prolongation,^10^ and it is suggested to avoid its use in patients with congenital Q‐T prolongation and to use caution using octreotide in conjunction with medications that prolong the Q‐T interval.^11^ In addition, hypertension has been reported in patients receiving octreotide with normalization of blood pressure after discontinuation of the drug. Two preterm infants receiving octreotide for enterocutaneous fistulas secondary to surgery for NEC experienced pulmonary hypertension with hypoxemia and increased oxygen requirement thought to be secondary to impaired vasodilatory mechanisms in the setting of neonatal respiratory distress syndrome.^12^

The use of octreotide in pediatric patients is complicated by the fact that it usually requires parenteral administration. Somatostatin analogues are available as an immediate release solution for injection, given via either subcutaneous (subQ) or intravenous (IV) routes, and a long‐acting injectable that is administered intramuscularly once per month (Table 1). There is an extended‐release oral preparation of octreotide that uses enteric coating and transient permeability enhancer to allow enteral absorption of the drug; however, use in children has not been described. This new medication may provide a future alternative to parenteral administration of octreotide.^14^ Lanreotide is another somatostatin analogue that is administered via deep subQ injection that has been used primarily for effects on GH release in adults with acromegaly. Its use has been documented in children.

**Table 1:** Somatostatin analogue formulations and standard dosing.

Dosage formulation	Route	General dose range	Notes
Octreotide Acetate	SubQ/intermittent IV dose	1–10 mcg/kg/dose every 8–12 h	Duration 6–12 h.
Octreotide acetate*	Continuous IV infusion	1–2 mcg/kg/h	Lower doses also used as subQ continuous infusion.
Octreotide delayed‐release capsules (Mycapsa)	PO	20 mg twice daily	Currently not used in pediatrics. Indicated for adults with acromegaly who have responded to and tolerated treatment with other preparations.
Octreotide LAR (Sandostatin LAR Depot)	IM	Dosing strategies vary. One approach is cumulative 31‐day subQ dose given once every 4 weeks with subQ overlap until steady state achieved	Steady state achieved after 3 injections administered at 4‐week intervals. Requires reconstitution.
Lanreotide (Somatuline Depot)	Deep SubQ	Every 4 weeks in doses of 60 to 120 mg	Provided in a prefilled syringe.

Abbreviations: IM, intramuscular; IV, intravenous; LAR, monthly long‐acting octreotide; PN, parenteral nutrition; PO, oral; SubQ, subcutaneous.

* Manufacturers caution against the use of octreotide PN because of concern for formation of a glycosyl octreotide conjugate that may decrease the product's efficacy. This has been questioned, and studies suggest that octreotide is not stabile in amino acid‐dextrose‐lipid emulsion beyond 24 h. There does appear to be a reduction in octreotide bioavailability that is ≤10%–15% at 48 h in both amino acid–dextrose and amino acid–dextrose‐lipid solutions.^13^

## CLINICAL APPLICATIONS FOR OCTREOTIDE

### GI bleeding

Many cases of pediatric GI bleeding resolve without the need for significant medical or endoscopic/surgical intervention. However, in patients in whom spontaneous resolution of bleeding does not occur or there is hemodynamic compromise, octreotide can be used. Octreotide acts by binding to receptors in blood vessels with a high affinity causing splanchnic vasoconstriction and a decrease in portal blood pressure,^6^ which can decrease bleeding. In children, acute upper GI bleeding (UGIB) originating above the ligament of Treitz, can be life‐threatening. In one study, the etiologies for 86 children with UGIB were gastric ulcer (32.5%), duodenal ulcer (26.7%), esophageal ulcer (10.5%), Mallory‐Weiss syndrome (8.1%), hemorrhagic erosive gastritis (5.8%), and esophageal varices (5.8%).^15^

The use of octreotide in pediatric UGIB has mostly been based on adult experience in patients with portal hypertension and bleeding from esophageal varices. Randomized controlled trials demonstrated the benefit of octreotide, in conjunction with endoscopic interventions, over ligation or sclerotherapy alone.^16^, ^17^ In most instances, octreotide acts as a temporary treatment before more definitive endoscopic surveillance and intervention. In one retrospective study, 21 children with portal hypertension during 35 episodes of UGIB and 12 children without portal hypertension with 14 episodes of UGIB were placed on continuous octreotide infusion. The rate of bleeding control achieved in patients with portal hypertension was 71% and in patients without portal hypertension was 80%.^18^ In another retrospective study of 18 episodes of UGIB secondary to portal hypertension in 13 children admitted to a pediatric intensive care unit, all episodes were controlled by octreotide infusion and patients showed a significant decrease in heart rate from the time of octreotide initiation to discontinuation (*P* < 0.02) and a significantly higher diastolic blood pressure and mean arterial pressure during the infusion as compared with prior (*P* < 0.05).^19^ There is a risk of rebleeding after discontinuation of octreotide therapy, and most gastroenterologists will wean the dose of octreotide over a few days.^18^, ^20^

Daily subQ octreotide or monthly long‐acting octreotide (OCT‐LAR) can be used in chronic GI bleeding; however, pediatric data are limited to small case series and retrospective reviews. One pediatric case series described successful use of daily subQ octreotide, dosed at 4–8 mcg/kg per day, in three pediatric patients with chronic cryptogenic GI bleeding, arteriovenous malformation, and portal hypertension. The patients had significant reduction in need for blood transfusions and had no significant side effects from the medication.^21^ In a study of nine pediatric patients with severe or refractory GI bleeding secondary to portal hypertension given long‐acting therapy with OCT‐LAR (2.5–20 mg intramuscular [IM] monthly), a reduction in the number of bleeding episodes was demonstrated in all the children and cessation of bleeding was observed in seven. In addition, two children listed for transplantation because of severe GI bleeding were removed from the transplant list.^9^

*Summary*: Although no trials exist in pediatric patients, there are ample adult data and a small body of pediatric data that suggest octreotide is effective at controlling bleeding in children with acute GI bleeds. The dosage suggested is 1–2 mcg/kg IV bolus followed by a continuous IV infusion of 1–2 mcg/kg/h for 2–5 days.^11^, ^18^ Careful attention should be paid to the risk of rebleeding after discontinuation of therapy. In addition, success has been reported with use of daily to twice daily subQ octreotide, as well as long‐acting IM octreotide, in cases of chronic GI bleeding.

### Motility

Octreotide decreases secretions in the GI tract and inhibits submucosal neurons via blockage of acetylcholine output. In patients with certain motility disorders, this can lead to improvement in nonpropulsive spastic activity.^22^ Evidence of effect in motility disorders is limited. Chronic intestinal pseudoobstruction (CIPO) is a motility disorder in which patients present with clinical features of bowel obstruction in the absence of a mechanical bowel obstruction. This disorder can be especially challenging to manage, as there is a lack of viable medical and surgical treatment options.^23^ In one study of children with chronic GI disorders, including CIPO, subQ octreotide was given to fasting patients and their motility was recorded via antroduodenal manometry. The authors found that octreotide induced phase 3 of the motor migrating complex, a marker of gastric antral and small bowel motility.^24^ In a clinical study of 16 pediatric patients with CIPO requiring total parenteral nutrition (PN) support, octreotide was started at a dose of 0.2–1 mcg/kg/day divided in two doses administered via central line over 60 min. This intervention allowed for 69% of the patients to tolerate increases in enteral nutrition support and some to discontinue PN.^25^

Our institution reported the use of octreotide in a 16‐year‐old male with a history of extensive subdural empyema with prolonged symptoms of pseudoobstruction, including emesis and feeding intolerance. Multiple attempts with motility agents and postpyloric feeding failed to improve his symptoms or nutrition status. Octreotide was initiated at a dosage of 100 mcg given subQ every 12 h and resulted in a precipitous drop in gastric output and resolution of nausea within 24 h. This allowed for tolerance of enteral nutrition advancement, and, ultimately, weight gain. Octreotide was discontinued after 13 days secondary to a side effect of pain at the injection site. The patient continued to do well and was discharged home on a full oral diet.^26^

Dumping syndrome is another disorder of motility that usually occurs as a side effect of gastric surgery and arises when large volumes of undigested food enter the small intestine quickly. Early dumping symptoms consist of abdominal pain, nausea, diarrhea, and the vasomotor symptoms of drowsiness and facial flushing. Late dumping symptoms are mostly related to reactive hypoglycemia.^27^ In children, this can be seen after fundoplication in which gastric motility is promoted^28^ either related to impaired postprandial fundic accommodation or vagal nerve damage.^29^ In cases in which dietary and lifestyle modifications do not improve symptoms, a somatostatin analogue should be considered. Several adult studies have shown that daily subQ injections prevent or significantly reduce symptoms of both early and late dumping.^30^, ^31^, ^32^ Published reports on use in pediatric patients with dumping is limited, with a case report demonstrating efficacy of octreotide in a pediatric patient with dumping post‐fundoplication at a dose of 2 mcg/kg/dose subQ twice per day.^11^

In a study of pediatric patients with chronic constipation and encopresis given octreotide during colonic manometry there were no significant changes in motility. The authors concluded that there is not a role for octreotide in recalcitrant constipation.^33^

*Summary*: Studies are limited on the use of octreotide for pediatric motility indications. Octreotide can be considered for those children with chronic intestinal pseudoobstruction or high gastric output causing feeding intolerance at a subQ dose of 0.5–1 mcg/kg every 12 h.^23^ Careful consideration should be given for its use in dumping syndrome. Although adult data exist, published pediatric cases are extremely limited. There is no role for the use of octreotide in patients with constipation.

### Lymphatics disorders

The lymphatic vessels are thin‐walled vessels that carry absorbed interstitial fluid from tissues to two major lymphatic ducts, the right lymphatic duct and the thoracic duct.^34^ Disruption, malformation, and increased hydrostatic pressure of the lymphatic vessels or venous system can lead to lymphatic disorders. Lymphatic disorders are rare in pediatric patients and include simple, localized lymphatic disorders causing fluid‐filled malformations and disorders affecting the flow of the lymphatic system.

Obstruction of lymphatic flow leads to chylous drainage in the pleural, pericardial, or peritoneal spaces. Chylous drainage can lead to respiratory distress, malnutrition, immunodeficiency, and a thrombogenic state, due to loss of important proteins and fat. Although rare, significant morbidity and mortality, as well as increased hospital costs, have been associated with chylous drainage in pediatric patients.^34^, ^35^, ^36^, ^37^ Prompt treatment of lymphatic flow disorders is necessary to potentially decrease morbidity and mortality.

The mechanism of somatostatin regarding chyle production and intraluminal pressure in the lymphatic system is not well understood. In theory, reduction in hepatic, portal, and splanchnic blood flow, decreases lymphatic circulation, which promotes healing of lymphatic vessels.^38^ Although little is known about the mechanism of action of somatostatin regarding chyle production and intraluminal pressure in the lymphatic system, inhibition of GI vasoactive peptides and stimulation of the autonomic nervous system are the proposed mechanisms of action.

#### Chylothorax

Pediatric chylothorax can be due to congenital causes and/or acquired causes following thoracic surgery due to direct injury to thoracic duct and/or other lymphatic vessels or due to increased venous pressures. Most studies to date have focused on the management of chylothorax postcardiac surgery given its incidence in 1%–9% of patients undergoing congenital heart surgery.^36^, ^39^, ^40^ The management and treatment of chylothorax aim to relieve respiratory symptoms and to prevent fluid reaccumulating by treating the etiology. Medical management of chylothorax in children, successful in >80% of reported cases, consists of dietary changes, including low‐fat diet with the addition of medium‐chain triglycerides (MCTs) or in severe cases nil per os with PN, and in some cases addition of somatostatin analogues.

Octreotide has been reported to be an effective treatment of chylothorax not responding to conservative medical management with nil per os status or PN. Octreotide was used for the first time in congenital chylothorax in 2003.^41^ Since the early 2000s, there have been multiple reports using octreotide for acquired and congenital chylothorax. In 2018, Bellini et al performed a systematic review on octreotide for congenital and acquired chylothorax in newborns. A total of 39 case reports were included. Octreotide decreased chylous drainage in 47% of patients. Although there was variation in octreotide regimens, the most common therapeutic route and dose were IV infusion starting at a dose of 1 mcg/kg/h and gradually increasing to 10 mcg/kg/h. Side effects were present in 12 of 84 patients (14.3%).^38^ In a single‐center study, Bui et al evaluated the efficacy of octreotide vs conventional therapy for postoperative chylothorax in pediatric patients in the cardiac intensive care unit. The patients who received octreotide had a longer duration of chest tube output compared with the conventional group (24 vs 9 days, *P* < 0.001); however, there was a significant and moderate correlation between octreotide and reduction of chylous output following initiation of octreotide (*R* ^2^ = 0.464, *P* = 0.021).^42^ The incidence of NEC appeared higher in the octreotide group, 12.5% vs 0%, though was not statistically significant (12.5% vs 0%, *P* = 0.287). Jenkinson et al performed the largest review of octreotide used for acquired chylothorax in children postcardiothoracic surgery in 2022. This review included 621 patients across 27 studies. Treatment efficacy was reported in 95% of studies. The authors concluded that octreotide efficacy is widely reported despite a lack of standardization on treatment initiation or definition of response to therapy. Of the 27 studies, side effects were limited and included disorders of glucose homeostasis, loose stools, and NEC, although no study reported a cessation of octreotide infusion in response to observed side effects.^43^

Several proposed algorithms consider the use of octreotide if chest tube drainage continues despite PN use or with PN if there is high‐volume chest tube output, >20 ml/kg/day.^44^, ^45^ The Pediatric Critical Care Consortium developed consensus recommendations for the management of postoperative chylothorax in pediatric congenital heart disease.^46^ Because of the varying evidence to support octreotide, the routine use of octreotide was not recommended and should only be considered if there is ongoing high‐volume chest tube output despite nil per os and PN. Despite multiple case reports and reviews with promising results with limited side effects, a consensus has not been reached on the optimal route, dose, or duration of therapy of octreotide for chylothorax in neonates and children.

#### Chylous ascites

Chylous ascites is rare in children, more so than chylothorax, and data on pathogenesis and management are sparse. There have been case reports of using octreotide successfully in chylous ascites in pediatric patients. In one case series, four patients were diagnosed with postoperative chylous ascites. Two patients treated with PN and octreotide had complete resolution of chylous drainage by day 7 vs 24–30 days in the patients not treated with octreotide.^47^ In another case series, there were two neonates with chylous ascites treated with octreotide. One patient had a decrease in drainage and the other did not. Maximum doses ranged from 4 mcg/kg/h to 8 mcg/kg/h.^48^

#### Intestinal Lymphangiectasia

Intestinal lymphangiectasia (IL) is a rare condition that occurs secondary to underlying lymphatic abnormalities and leads to the loss of lymphatic contents into the intestinal lumen. This condition can be primary (PIL) or can be secondary to heart surgery, neoplasm, or infection.^49^ The resulting clinical picture is that of a protein‐losing enteropathy (PLE) with the clinical manifestations of hypoalbuminemia, including generalized edema, pleural and pericardial effusions, ascites, abdominal distention, diarrhea,^50^ and hypogammaglobulinemia. The intestinal lymphatic system plays a crucial role in the absorption of long‐chain triglycerides (LCTs). First‐line management of PIL is restriction of dietary LCTs in conjunction with the use of MCT‐rich supplements. This strategy is thought to reduce intestinal lymph flow and decrease intestinal protein losses.^51^

A retrospective review of children with IL treated between 1999 and 2008 at a single center described the effect of octreotide on disease outcomes and drug safety. All patients were also managed with a high‐protein, low‐fat diet with MCT supplements. Six children with PIL were dosed with octreotide, 15–20 mcg/kg subQ twice per day, with a duration that ranged from 3 to 37 months. All patients had a decrease in their dependency on serum albumin infusions, and three patients were able to maintain normal serum albumin levels. Stool frequency decreased in all the patients after starting octreotide treatment. The only reported side effect was acute pancreatitis (AP) after 37 months of receiving therapy, which resolved with supportive treatment. The authors concluded that octreotide was safe and that it may help to maintain serum albumin levels, improve clinical findings, and decrease the requirement of serum albumin infusions in refractory cases of primary IL.^52^

*Summary*: Although there are a lack of prospective and randomized controlled trials supporting octreotide use for chylous drainage in children, studies highlight octreotide (dose 1–10 mcg/kg/hr via continuous IV infusion) may be a safe option with minimal side effects for treatment of refractory acquired and congenital chylothorax and chylous ascites. A small case series suggests that intestinal lymphangiectasia can be treated with octreotide at a dosage of 15–20 mcg/kg subQ twice per day.

### Pancreatic disorders

AP and chronic pancreatitis are estimated to occur at rates of 1 in 10,000 and 2 in 100,000, respectively.^53^, ^54^ AP in children requires at least 2 of the following: abdominal symptoms consistent with AP; serum amylase or lipase values three times the upper normal level; and imaging findings consistent with AP.^55^ A subset of pediatric patients with AP, 13%–30%, are classified as moderately severe or severe. Moderately severe pancreatitis is characterized by the presence of transient organ failure, local complications, or exacerbation of comorbid disease.^56^ The most common local complications are acute perihepatic fluid collections and pancreatic pseudocysts.^57^

The role of octreotide in pediatric AP has not been established, and adult data are conflicting. A randomized, double‐blind, multicenter trial of octreotide in moderate to severe AP in adults (*n* = 302) showed no difference among patients receiving placebo, or three times daily injections of 100 or 200 mcg on mortality, development of complications, duration of pain, surgical interventions, or length of stay.^58^ However, a case‐control prospective randomized study (*n* = 50) in acute severe pancreatitis in adults showed decreased rates of sepsis (24% vs 76%, *P* = 0.0002), acute respiratory distress syndrome (28% vs 56%, *P* = 0.04), mortality (4% vs 16%), and a shorter hospital stay (20.6 vs 33.1 days, *P* = 0.04).^59^ In a 2021 report of pediatric patients with acute or chronic pancreatitis enrolled in INSPPIRE, only 2% of patients (*n* = 456) had received octreotide as a treatment modality.^60^

Octreotide has been used to prevent reoccurrence of asparaginase‐induced AP. Small pediatric case series demonstrate that prophylactic octreotide dosed as a continuous infusion (*n* = 7)^61^ or subQ (*n* = 3)^62^ was effective at preventing reoccurrences and allowed continued use of asparaginase in cancer management.

In a retrospective study of adult patients with pancreatic injury, patients treated with octreotide did not show a decrease in the incidence or severity of abdominal complications when stratified by pancreatic injury severity. It was observed that patients treated with octreotide had a higher rate of fistula formation (48% vs 40%), longer duration of fistula drainage, and longer hospital stay compared with untreated patients.^63^ It is not recommended to initiate prophylactic octreotide in adults with pancreatic injury to prevent pancreatic fistula formation.^64^

Octreotide may have a role in the treatment of local complications, such as pancreatic fistula, pseudocyst, and intra‐abdominal abscesses. A 19‐month‐old boy with pancreatic pseudocyst after blunt abdominal trauma had resolution of pseudocyst after receiving octreotide at a dosage of 1 mcg/kg/h continuous intravenous infusion for 24 h, followed by 2.5 mcg/kg/dose every 12 h subQ for 11 days.^65^ Two pediatric patients had resolution of pancreatic pseudocyst within 5 weeks while being treated with octreotide 2.5 mcg/kg subQ daily, bowel rest, and PN.^66^ A 5‐year‐old girl with the rare complication of ruptured pancreatic pseudocyst occurring 27 days after trauma was successfully managed nonsurgically with octreotide.^67^ A 10‐year‐old boy had rapid regression of a posttraumatic pancreatic pseudocyst on octreotide.^68^ Octreotide has been used in the management of high‐output traumatic pancreatic fistulae in three pediatric patients. The treatment was well tolerated without side effects and resulted in a dramatic decrease in the amount of fistula drainage within the first 24 to 48 h.^69^ A 7‐month‐old infant with pancreatitis and ascites was managed successfully with subQ octreotide and external drainage of a pseudocyst.^70^

Historically, octreotide was used as prophylaxis for post–endoscopic retrograde cholangiopancreatography (ERCP). During this procedure, the bile ducts and pancreatic ducts are accessed to diagnose and treat a variety of pancreaticobiliary pathology. The most common adverse event of ERCP is post‐ERCP pancreatitis, occurring after ~8%–15% of adult procedures.^71^ A 2007 meta‐analysis showed that octreotide was ineffective in preventing post‐ERCP pancreatitis.^72^ There are no data on the use of octreotide to prevent pediatric pancreatitis post‐ERCP, as most studies are done on adults. Pediatric ERCPs represent only a small fraction of the total number of ERCPs completed; in one center 4% (120 of 3000) of procedures done annually.

*Summary*: There are few pediatric data on the use of octreotide in patients with acute or chronic pancreatitis. Small case series suggest octreotide may help prevent a recurrence of asparaginase‐induced pancreatitis in patients receiving chemotherapy. There appears to be a role for use of octreotide in pediatric patients with the development of local complications of AP, although no pediatric trials have been conducted. Adult data do not support the usage of octreotide to prevent post‐ERCP pancreatitis.

### High secretory output

Octreotide inhibits prosecretory substances such as serotonin, gastrin, secretin, and VIP and motilin. It has been used in GI disorders associated with high output, including high‐output ileostomies, chronic diarrhea, diarrhea associated with chemotherapy and graft vs host disease (GVHD), vomiting/obstruction, and neuroendocrine tumors.

In pediatrics, the definition of a high‐output ileostomy (HOI) has not been standardized, and cutoffs from 20 to 50 ml/kg/day or a maximum of 1500 ml/day have been used in different studies.^73^ Children with HOI are at risk for dehydration, electrolyte abnormalities, vitamin and mineral deficiencies, and poor growth. When dietary modifications do not prove adequate, medical therapy should be initiated. In three children with HOI, significant decreases were seen with subQ octreotide 6–6.5 mcg/kg/day in two doses (88/43 ml/kg/day, 57/22 ml/kg/day, and 120/40 ml/kg/day). It was also noted that after therapy was discontinued, the output increased again.^74^, ^75^ Additionally, there is a paucity of quality adult data in the benefits of somatostatin analogues in HOI. One recent prospective randomized case‐control study was prematurely stopped due to low number of patients; no difference seen between patients receiving or not receiving octreotide 1900 ± 855.7 ml and 1728.6 ± 845.5 ml, respectively (*P* = 0.2).^76^

Reports of the use of octreotide in idiopathic chronic diarrhea of infancy date back to the 1980s.^77^, ^78^ A pediatric patient with microvillus inclusion disease had significant decline in stool volume, from 275 ml/kg/day to 161 ml/kg/day, as well as reduction in stool electrolytes and an increase in urine output while being treated with octreotide. Over the course of a 9‐month period, no side effects or impaired growth were noted.^79^ There are no prospective studies on the use of octreotide or other somatostatin analogues in pediatric patients. A study of adults with chronic idiopathic diarrhea (*n* = 33) who received lanreotide subQ at days 0, 28, and 56 (40) found a reduction by >50% in stool frequency (5.6 to 2.3 per day) in 51.5% of patients at day 56.^80^

Several clinical studies in patients with short bowel syndrome have reported a reduction of intestinal output in patients taking octreotide compared with controls. Long‐term studies of adults with variation of intestinal length and output have shown reduction in intestinal output between 16% and 72%.^81^ However, the use of octreotide in short bowel syndrome has been limited because of concerns about potential adverse effects on intestinal adaptation related to its inhibition of a number of GI‐trophic hormones.^82^

Octreotide is FDA‐approved for adults with severe diarrhea secondary to carcinoid tumors, and severe diarrhea secondary to VIP‐secreting tumors. Although much less common, these tumors can present in pediatric patients, and use has been documented in infants as young as 8 weeks with elevated VIP concentrations and secretory diarrhea.^83^

Diarrhea is common in pediatric oncology patients secondary to chemotherapy or related to GVHD in allogeneic hematopoietic stem cell transplant patients. A retrospective study evaluated the efficacy and safety of octreotide in children for the treatment of chemotherapy‐induced diarrhea (CID) (*n* = 25) or GVHD‐induced diarrhea (*n* = 11). Overall, results showed that octreotide exhibited a 92% efficacy in treating CID and 45% efficacy in GVHD‐induced diarrhea.^84^

In addition to high stool and ileostomy output, octreotide has been used for persistent high‐volume emesis. In two pediatric case reports, octreotide was used in the setting of bowel obstruction. In one case, octreotide was administered to a 12‐year‐old boy receiving palliative care for peritoneal dissemination of colon cancer resulting in partial bowel obstruction. After one week of intravenous octreotide, the patient's nausea improved, vomiting ceased, and appetite increased.^85^ Another case report demonstrated cessation of bilious emesis in a 12‐year‐old girl with malignant obstruction of the distal duodenum while receiving IV octreotide.^86^

Enterocutaneous fistulas (ECFs), abnormal connections between the GI tract and the skin, which allow intestinal contents to leak through an opening in the skin, are a rare surgical complication in children, most often related to surgical intervention for NEC, intestinal atresia, or volvulus.^87^ The overall goal of treatment for ECFs is to close the fistula early to help decrease rates of sepsis, malnutrition, dehydration, and electrolyte disturbances.^88^ The overall healing rate of ECFs with conservative management is 54%, with time to healing ranging from 6 to 107 days.^89^, ^90^ A review of octreotide in ECFs included nine pediatric patients with ECFs treated with octreotide. The authors concluded that there is a role for octreotide in ECFs and highlighted early initiation of therapy with intermediate to high dosages (6–300 mcg/kg/day), and continuous IV administration with a gradual withdrawal to avoid rebound effects reported with abrupt discontinuation or intermittent dosing.^87^ It may be most useful in patients with proximal fistulas for which output is very high and surgical intervention is more commonly needed.

*Summary*: No trials exist on the use of octreotide in a variety of high‐output states in children; evidence is limited to case reports and series. In children with high‐output ileostomy, a dosage of 6–6.5 mcg/kg/day in two doses can help decrease output. In chronic noninfectious diarrhea, adult studies and pediatric case reports support the use of octreotide although no dosing has been established. Octreotide may be beneficial in diarrhea related to GVHD or chemotherapy. There is concern octreotide may hinder adaptation in patients with short bowel syndrome given octreotide's inhibition of GI‐trophic hormones. There are case reports of octreotide usage in persistent high‐volume emesis secondary to malignant obstruction. Case series suggest the octreotide can be started to help hasten the closure of enterocutaneous fistulas at doses of 6–300 mcg/day via IV infusion.

## CONCLUSION

There are no prospective randomized trials on the use of octreotide in pediatric patients. However, the use of octreotide in pediatric patients has been described in many GI disorders and disorders related to the loss of vital proteins and across the lymphatic system (Table 2). Octreotide is generally considered safe, but clinicians should be aware of potential side effects, including bradycardia, hyperglycemia, abdominal discomfort with nausea/diarrhea, hypothyroidism, and cholelithiasis. Prospective studies and registries of children treated with octreotide should be considered to help guide clinicians on indications and dosage.

**Table 2:** Indications, evidence, and dosage for octreotide in pediatric patients.^91^

Indication	Quality of evidence	Dosage
GI bleeding	Case reports^11^, ^21^ Retrospective review^4^, ^18^	IV: For acute UGIB. Loading dose of 1–2 mcg/kg followed by continuous infusion of 1–2 mcg/kg/h. Adult dosing is 50 mcg bolus followed by 50 mcg/h. Can be increased every 8 h if there is no decrease in bleeding. Once bleeding has ceased can decrease dosage by 50% every 12 h and stop when at 25% of the initial dose. SubQ: For chronic GI bleeding. 4–8 mcg/kg/day in 1–2 divided doses IM: Sandostatin LAR Depot for chronic GI bleeding. 2.5–20 mg IM monthly
CIPO	Case studies^25^	IV: 1 mcg/kg/day divided in two doses given over 60 min
Dumping syndrome	Case reports^11^	SubQ: 2 mcg/kg/dose twice daily
Chylothorax/chyloperitoneum	Case reports^92^, ^93^, ^94^, ^95^ Case studies^96^, ^97^ Retrospective reviews^98^ Systematic reviews^38^, ^99^, ^100^, ^101^	IV: 0.5 to 3 mcg/kg/h as initial infusion; increased by 1 mcg/kg/h every 24 h until desired response. Maximum dosage 10 mcg/kg/h. Once chylothorax resolved decrease infusion by 1 mcg/kg/h every 24 h. SubQ: Initial dosages of 10 mcg/kg/day in three divided doses. Dose titrated upward by 5–10 mcg/kg/day every 3–4 days to effective range (10–40 mcg/kg/day). After 3 days of chyle output <10 ml/day titrate down by decreasing 10 mcg/kg/day
IL	Case series^52^	SubQ: 15–20 mcg/kg/dose twice daily
Pancreatitis, management of complications	Case reports^68^, ^102^	IV: 1.5 mcg/kg/h continuous infusion SubQ: 2 mcg/kg/dose every 6 h increased up to 20 mcg/kg/dose for recurrence
Pancreatitis, prevention of pancreatitis secondary to asparaginase	Case reports^61^, ^62^	SubQ: 2.5 mcg/kg/dose once daily IV: 2.5–5 mcg/kg/day continuous infusion begun 24 h before the first day of asparaginase and continued through treatment and for 10 days after last dosage of asparaginase
Diarrhea: High‐output secretory diarrhea, HOI, diarrhea related to chemotherapy or GVHD, VIP‐secreting tumors	Case reports^74^, ^75^, ^78^, ^79^, ^103^ Retrospective reviews^84^	SubQ: Preferred method of delivery. 2–10 mcg/kg/day divided every 8–12 h. Titrate up to clinical response. IV: Used in patients with inadequate response to SubQ dosing. Can be given intermittently at 2–10 mcg/kg/day divided every 8 h. Via continuous infusion initiate at 0.25–1 mcg/kg/h and titrate up every 24–48 h to desired effect
High‐output emesis secondary to malignant obstruction	Case reports^85^, ^86^	IV: 2 mcg/kg/h SubQ: 100 mcg/dose given once for palliative care
ECF	Case reports^87^, ^104^, ^105^, ^106^	SubQ/IV: Large variations in dosing. Most commonly, 1–1.5 mcg/kg/day gradually increased to a maximum dosage 3–6 mcg/kg/day

Abbreviations: ECF, enterocutaneous fistula; GI, gastrointestinal; GVHD, graft vs host disease; HOI, high‐output ileostomy; IM, intramuscular; IL, intestinal lymphangiectasia; IV, intravenous; LAR, monthly long‐acting octreotide; PO, oral; SubQ, subcutaneous; UGIB, upper GI bleed; VIP, vasoactive intestinal peptide.

## AUTHOR CONTRIBUTIONS

Candi Jump, Bailey Dunn, Megan Foxe, and Kathy Hunter Sprott equally contributed to the conception and design of the paper. All authors drafted the manuscript, critically revised the manuscript, agree to be fully accountable for ensuring the integrity and accuracy of the work, and read and approved the final manuscript.

## CONFLICT OF INTEREST STATEMENT

None declared.
